# A homozygous *PIGN* missense mutation in Soft-Coated Wheaten Terriers with a canine paroxysmal dyskinesia

**DOI:** 10.1007/s10048-016-0502-4

**Published:** 2016-11-28

**Authors:** Ana L. Kolicheski, Gary S. Johnson, Tendai Mhlanga-Mutangadura, Jeremy F. Taylor, Robert D. Schnabel, Taroh Kinoshita, Yoshiko Murakami, Dennis P. O’Brien

**Affiliations:** 1 Department of Veterinary Pathobiology, College of Veterinary Medicine, University of Missouri, Columbia, MO USA; 2 Division of Animal Sciences, College of Agriculture, Food and Natural Resources, University of Missouri, Columbia, MO USA; 3 Informatics Institute, University of Missouri, Columbia, MO USA; 4 Department of Immunoregulation, Research Institute for Microbial Diseases, and Laboratory of Immunoglycobiology, WPI Immunology Frontier Research Center, Osaka University, Osaka, Japan; 5 Department of Veterinary Medicine and Surgery, College of Veterinary Medicine, University of Missouri, Columbia, MO 65211 USA

**Keywords:** Glycosylphosphatidylinositol, Multiple congenital anomalies-hypotonia-seizures syndrome-1 (MCAHS1), Phosphatidylinositol glycans, Ethanolamine phosphate transferase-1

## Abstract

Hereditary paroxysmal dyskinesias (PxD) are a heterogeneous group of movement disorders classified by frequency, duration, and triggers of the episodes. A young-adult onset canine PxD has segregated as an autosomal recessive trait in Soft-Coated Wheaten Terriers. The medical records and videos of episodes from 25 affected dogs were reviewed. The episodes of hyperkinesia and dystonia lasted from several minutes to several hours and could occur as often as >10/day. They were not associated with strenuous exercise or fasting but were sometimes triggered by excitement. The canine PxD phenotype most closely resembled paroxysmal non-kinesigenic dyskinesia (PNKD) of humans. Whole genome sequences were generated with DNA from 2 affected dogs and analyzed in comparison to 100 control canid whole genome sequences. The two whole genome sequences from dogs with PxD had a rare homozygous *PIGN:c.398C* > *T* transition, which predicted the substitution of an isoleucine for a highly conserved threonine in the encoded enzyme. All 25 PxD-affected dogs were *PIGN:c.398T* allele homozygotes, whereas there were no *c.398T* homozygotes among 1185 genotyped dogs without known histories of PxD. *PIGN* encodes an enzyme involved in the biosynthesis of glycosylphosphatidylinositol (GPI), which anchors a variety of proteins including CD59 to the cell surface. Flow cytometry of *PIGN*-knockout HEK239 cells expressing recombinant human *PIGN* with the *c.398T* variant showed reduced CD59 expression. Mutations in human PIGN have been associated with multiple congenital anomalies-hypotonia-seizures syndrome-1 (MCAHS1). Movement disorders can be a part of MCAHS1, but this is the first PxD associated with altered GPI anchor function.

**Electronic supplementary material:**

The online version of this article (doi:10.1007/s10048-016-0502-4) contains supplementary material, which is available to authorized users.

## Introduction

The human paroxysmal dyskinesias (PxD) are a heterogeneous group of diseases characterized by episodes of abnormal involuntary movements [1]. The episodes may last <1 min or continue for many hours. Episode frequency may vary from <1/year to >100/day. The abnormal movements can be dystonia, chorea, athetosis, or ballism, either singly or in various combinations [2–4]. Many PxD episodes are initiated by a recognized trigger, and the type of trigger has served as a means of subclassifying PxD [1, 2, 5]. In paroxysmal exertional dyskinesia (PED), the episodes are triggered by sustained exercise. Sudden movements trigger the episodes in paroxysmal kinesigenic dyskinesia (PKD). In paroxysmal non-kinesigenic dyskinesia (PNKD), stress, fatigue, and hunger can trigger episodes in some patients, but the most consistent triggers are alcohol or caffeine consumption. Other factors considered in classification are age of onset, frequency and duration of the episodes, and response to therapy [1, 2, 5–7].

PxD may be secondary to a specific etiology such as encephalitis or stroke [8] or idiopathic. The latter can be sporadic or familial, usually with an autosomal dominant inheritance [5]. Mutations in three genes, *SLC2A1*, *PRRT2,* and *PNKD* (also referred to as *MR-1*), are well-recognized causes of PxD [5, 9–13]. *SLC2A1* encodes glucose transport protein 1 (GLUT1), which facilitates glucose transfer across the blood-brain barrier. Most patients with heterozygous *SLC2A1* mutations exhibit GLUT1-deficiency syndrome characterized by low CSF-to-blood glucose ratios together with epilepsy, developmental delay, microcephaly, ataxia, and PxD [14]. A minority of patients with heterozygous *SLC2A1* mutations develop isolated PED [11, 15–17]. *PRRT2* encodes proline-rich transmembrane protein 2, which interacts with SNAP25 and thereby influences the release of glutamate or other neurotransmitters [18]. Heterozygous *PRRT2* mutations have been found in patients with several paroxysmal neurologic disorders including PKD [19]. Many PNKD patients carry heterozygous mutations in *PNKD* [5, 6, 13]. This gene encodes a protein that may suppress synaptic vesicle release by interacting with RIM1 and RIM2 [20]. Although *SLC2A1*, *PNKD*, and *PRRT2* harbor the majority of the causal mutations for the genetically defined cases of PxD [5], a few patients have been reported to carry potentially causal mutations in other genes such as *KCNMA1* [21] and *PDHA1* [22]. Efforts to identify the mutations responsible for PxD in other families have not yet been successful [6].

Dogs can also develop PxD [23]. Published reports have provided clinical descriptions of primary canine PxD (cPxD) or cPxD-like diseases in several breeds including the Bichon Frise [24], Border Terrier [25], Cavalier King Charles Spaniel [26], Chinook [27], Doberman Pinscher [28], English Bulldog [29], Scottish Terrier [30], and Soft-Coated Wheaten Terrier (SCWT) [31]. Unlike the human primary PxD that are almost always dominant traits, the cPxD usually have a recessive mode of inheritance. The cPxD that occurs in the Cavalier King Charles Spaniel is typically precipitated by exercise [26]. It is caused by a homozygous 16-kb microdeletion that includes the first three exons of *BCAN* [32, 33], which encodes the extracellular-matrix protein brevican. The molecular genetic causes of the other cPxD have not yet been reported. We now present evidence that SCWT with cPxD have a deficiency in the biosynthesis of glycosylphosphatidyinositol (GPI) anchors due to a homozygous missense mutation in *PIGN*, the gene that encodes a GPI synthesis enzyme, GPI ethanolamine phosphate transferase-1.

## Methods

### Animals

**Fig. 1:**
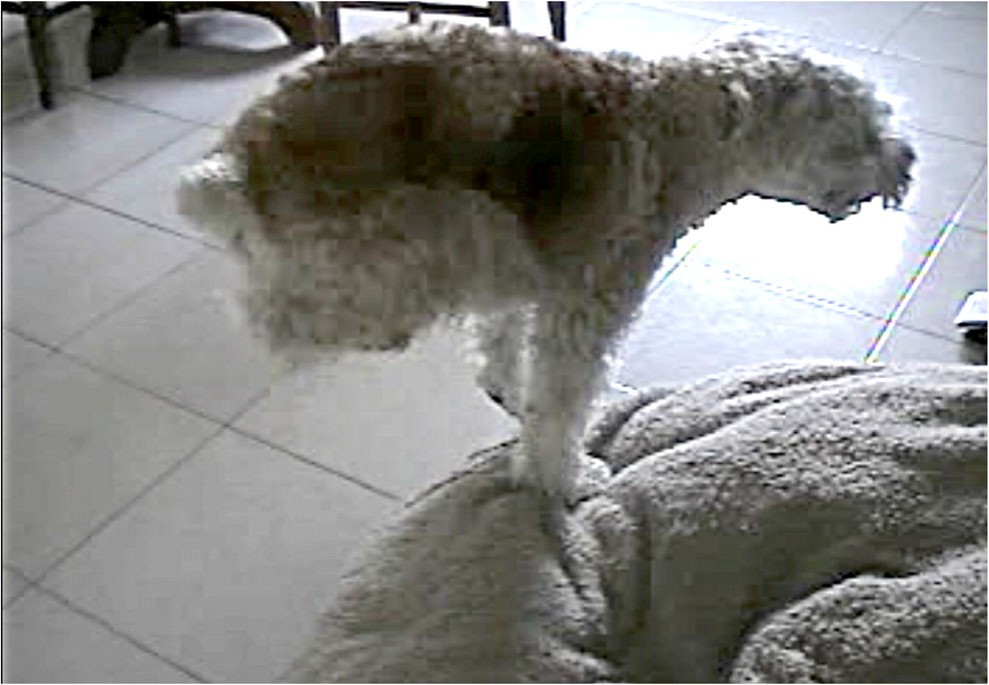
A 4-year-old, SCWT during a dyskinesia episode showing hyperflexion of the hind limbs that elevated both limbs off the ground simultaneously

Medical records and available videos of dyskinesia episodes were reviewed for 22 SCWT and 3 “Whoodles” (SCWT × Poodle crosses) with cPxD. The median age of onset of signs was 2.25 years. Males and females were affected equally. All dogs had normal neurologic exams between the episodes. Episode duration ranged from several minutes up to >4 h, and the frequency of episodes ranged from 1 every few days to >10/day. In six cases, stress, excitement, or loud noises were thought to precipitate some attacks, but a clear trigger was not apparent in most instances. No dogs had a history of ingestion of alcohol or caffeine. Typical episodes consisted of rapid flexion and extension of the hind limbs with varying degrees of truncal dystonia (Supplementary Video). Most frequently, the flexion alternated irregularly between limbs, but sometimes both hind limbs were off the ground simultaneously (Fig. 1). In severe episodes, the front limbs were also affected. No consistent improvement in episodes was reported with anti-epileptic drugs or benzodiazepines. The signs typically worsened with age, and six dogs were euthanized within 2 years after onset of signs due to the increasing severity of the episodes. MRIs were obtained from five affected dogs, and postmortem exams were performed on four dogs, but no abnormalities were identified in any of the examined brains.

### Molecular genetics

DNA was collected from 25 cPxD-affected dogs. In addition, we used archived DNA samples from 665 cPxD-free SCWT, 388 Poodles, and 132 randomly selected dogs from other breeds to validate the identified candidate causal variant. Whole genome sequences were generated with DNA from two different cPxD-affected SCWT. PCR-based DNA libraries were used for the first whole genome sequence as previously described [34]. PCR-free DNA libraries and slightly modified procedures [35] were used for the second. Both data sets were deposited in the Sequence Read Archives (accession numbers SRX863100 and SRX863101). Previously described procedures were used for trimming adaptor sequences from the sequence reads, eliminating duplicate reads, error correction, and alignment to the CanFam3.1 canine reference genome sequence [34, 36]. Sequence variant information was uploaded to a custom PostgreSQL database, which also contained sequence variant information from the aligned whole genome sequences of normal canids or dogs with diseases other than cPxD. These other sequences served as controls for the current analysis. The University of Missouri DNA Core Facility generated 43 of the control dog genome sequences; the rest were provided by individuals at other institutions as indicated in the “Acknowledgements.”

PCR primers *5′-GCTATACTAATATTTCACCGTTC-3′* and *5′-AAAATATAGTAAGTAATACACAA-3′* were used to amplify a segment of genomic DNA containing candidate sequence variant *PIGN:c.398C* > *T*, identified from the whole genome sequences, so that the sequence variant could be verified by direct automated Sanger sequencing. An allelic discrimination assay [37] was used to genotype DNA samples from individual dogs at this sequence variant. PCR primers for this assay were *5′-TGTGGGATTTGATTCTCTTATTAATG-3′* and *5′-TCAATGACTCTTACCTTTGGCAAACATA-3′*. The competing probe sequences were *5′-VIC-CCAGCTCCAT* ***G*** *TGTACC-MBG-3′* (reference *C* allele) and *5′-FAM-CCAGCTCCAT* ***A*** *TGTACC-MBG-3′* (variant *T* allele).

*PIGN-*knockout HEK293 cells were generated and transfected as previously described [38] with human wild-type or mutant (T133I, T133S, or T133V) *PIGN* complementary DNA (DNA) cloned into pME, a strong SRα promoter-driven expression vector, or pTK, a medium TK promoter-driven expression vector. These PIGN constructs had an HA epitope tag at the N-terminus. Transfection efficiencies were determined by the luciferase activity of cell lysates. After 3 days of incubation, restoration of the cell-surface expression of CD59 was evaluated by flow cytometry [38]. Levels of expressed wild-type and mutant HA-tagged PIGN in pME vector-transfected cells were determined by western blotting using an anti-HA antibody.

## Results

### Molecular genetics

Partial pedigree information was available for some of the affected dogs. Because no records indicated that the sires or dams of any of the affected dogs also exhibited cPxD, we assumed an autosomal recessive inheritance in our data analysis.

The first generated whole genome sequence from a cPxD-affected dog had a 21-fold average coverage of the reference genome and contained 6.9 million potential sequence variants (homozygous or heterozygous differences from the reference canine genome sequence). When this sequence variant information was first added to a custom PostgreSQL database, it also contained sequence variant information from eight control canine WGSs. The SCWT sequence variants were filtered to identify only those that fit three criteria: (1) they were predicted to alter the amino acid sequence of the gene product (including those that alter exon-splicing signals), (2) they were homozygous in the cPxD-affected dog’s whole genome sequence, and (3) they were absent from the control whole genome sequences. In the initial analysis, 418 sequence variants met these criteria. However, no plausible candidate sequence variants were identified from a review of the published biological functions and disease associations for the 302 genes that harbored these sequence variants.

As additional control whole genome sequences were added to the database, the number of homozygous coding variants unique to the affected SCWT decreased. Nonetheless, we were still unable to recognize a potential cPxD-causing sequence variant. Thus, we generated a whole genome sequence from another cPxD-affected dog. This sequence had 18-fold average reference genome coverage and contained 5.5 million potential sequence variants. By the time that the variants from the second cPxD-affected SCWT were included in our database, it contained variant data for 100 canid sequences that could be considered to be controls for the SCWT PxD. At this time, the whole genome sequence for the first case SCWT contained 230 variants in 162 different genes that met our criteria for candidate causality (Supplementary Table 1a). The whole genome sequence for the second SCWT PxD case had 65 candidate variants in 54 different genes (Supplementary Table 1b). The higher number of variants from the first case is attributable to the many false positives among the filtered variants from the early PCR-amplified libraries. The only sequence variant common to both animals was *PIGN:c.398C* > *T*, which predicted a p. T133I substitution in PIGN, the gene product. Direct automated Sanger sequencing of PCR amplicons produced with primers spanning the *PIGN:c.398C* > *T* variant confirmed that both of the SCWT with cPxD were *PIGN:c.398T* homozygotes.

All 25 of our DNA samples from cPxD-affected dogs tested homozygous for the variant *PIGN:c.398T* allele. Twenty two of these affected homozygotes were SCWT, and the other three were Whoodles. Whoodles are marketed as the product of F_1_ crosses between purebred SCWT and purebred Poodles. If this was indeed the origin of the three *c.398T* homozygous Whoodles, it would require that the *PIGN:c.398T* allele had been segregating in both breeds. Thus, we genotyped individual representatives from each breed at *PIGN:c.398C* > *T*. Fifteen of the 682 control SCWT were heterozygotes; the remaining 667 were homozygous for reference allele *c.398C.* All 388 of the genotyped Poodles were *PIGN*:*c.398C* homozygotes, as were all 132 randomly selected representatives of 92 other breeds. Although the *PIGN:c.398T* allele was not present in the Poodles that we genotyped, it could still be carried by other Poodles. Nonetheless, an alternative and more likely explanation for the origin of the three homozygous Whoodles is that they resulted from a Whoodle to SCWT backcross or from two Whoodle parents.

### Flow cytometry

**Fig. 2:**
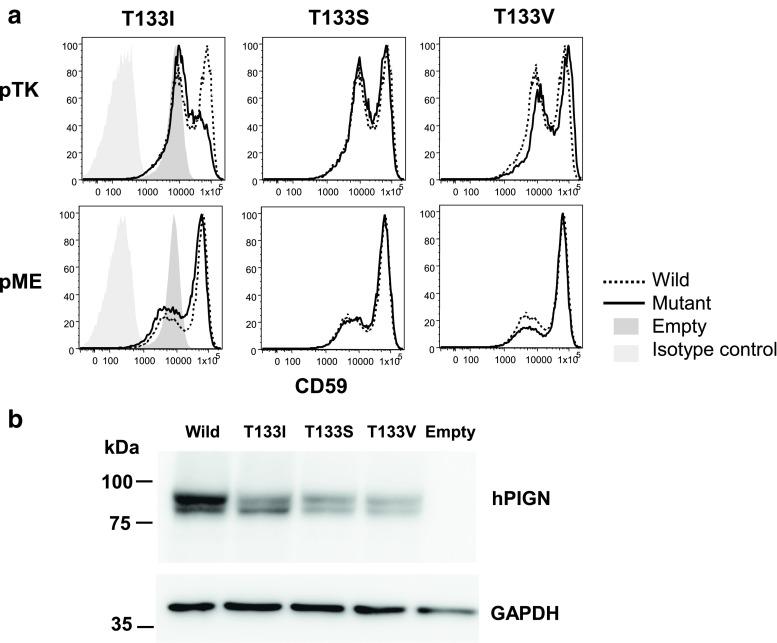
Functional analysis of *PIGN* mutants in *PIGN*-knockout HEK293 cells. **a** Restoration of cell-surface expression of GPI-anchored protein CD59 on *PIGN*-knockout HEK293 cells after transfection. HA-epitope-tagged, wild-type T331I, T331S, and T331V human *PIGN* cDNAs were transfected with a medium promoter-driven pTK vector (*top*) or a strong promoter-driven pME vector (*bottom*). Three days later, CD59 levels were assessed by flow cytometry. **b** Western blotting analysis of wild-type T331I, T331S, and T331V human PIGN. *PIGN*-knockout HEK293 cells that were transfected with HA-epitope-tagged, wild-type and mutant *PIGN* cDNAs in pME were analyzed 3 days later by western blotting using anti-HA antibody. *GAPDH* glyceraldehyde 3-phosphate dehydrogenase, a loading control

The biological activities of mutant PIGN isoforms were assessed by transiently transfecting corresponding cDNAs into *PIGN-*knockout HEK293 cells and measuring the restoration of GPI-anchored CD59 surface expression [38, 39]. Flow cytometry of cells which were transfected with a medium promoter-driven expression vector revealed comparable restoration of CD59 cell-surface expression by *PIGN-*knockout HEK293 cells 3 days after transient transfection with wild-type human *PIGN* cDNA or with recombinant human cDNAs designed to encode PIGN:p. T133S or PIGN:p. T133V isoforms. However, transient transfection with recombinant human cDNAs designed to encode PIGN:p. T133I restored only 60–70% of the cell-surface CD59 that was produced by the wild-type cDNA (Fig. 2a). The same T331I PIGN cDNA, when expressed with a strong promoter-driven vector pME, nearly fully restored CD59 (Fig. 2a). Western blotting revealed comparably expressed protein levels of T331I and wild-type PIGN (Fig. 2b). It is therefore concluded that the T331I mutation causes a reduction in specific activity rather than stability of PIGN.

## Discussion

We studied a cPxD originally described in SCWT in 2004 [31], which shared features of human PxD. All the affected dogs were normal on examination between episodes. The movements appeared to be involuntary and could not be disrupted. The dogs remained conscious during the episodes, and except for severe episodes, the dogs continued to walk, suggesting that the movements were semi-purposeful. Some of the movements in this cPxD were the sustained, abnormal postures of a dystonia [40]. The more rapid movements in cPxD are hyperkinetic movements, but they are difficult to place into one of the human categories of chorea, ballism, or athetosis [41]. The various forms of human PxD can be subclassified as a PED, PKD, or PNKD [1–4, 6, 7]. The owners of dogs with cPxD never mentioned exercise or fasting as triggers of dyskinesia episodes as occurs with PED in humans [42]. Determining whether a sudden movement triggered the episodes as in PKD in humans [7] was difficult since the owners typically were not closely observing their dogs at the onset of the episodes. Compared to the durations and frequencies of episodes of dyskinesia in human PKD patients [7], the episodes in dogs with *PIGN*-associated cPxD lasted longer (minutes to hours) and occurred less frequently (several/day to every few weeks), more like PNKD in humans [6]. Stress and excitement, which trigger episodes in most *PNKD* associated with PNKD in humans [6], were reported to trigger the episodes in some dogs, although in most cases, no trigger could be identified. While alcohol or caffeine ingestion are the most consistent triggers of human PNKD associated with *PNKD* mutations [6], dogs rarely ingest alcohol and methylxanthines are toxic to dogs [43], so whether they would trigger episodes in dogs is unknown. PKD patients either require no treatment or respond to anti-epileptic drugs, and PNKD patients often respond to benzodiazepines [2, 5–7], while SCWT with cPxD showed a poor response to these drugs. The median age of onset in dogs was 2.25 years, a young but fully mature adult dog, which would be older than the equivalent average age of onset in humans of *PNKD* associated with PNKD or PKD and more comparable to “atypical” PNKD not associated with *PNKD* mutations [6, 7]. Thus, even though there are differences, the dyskinesia episodes in *PIGN*-associated cPxD most closely resemble those of human PNKD patients.

To identify the molecular genetic cause of cPxD, we generated whole genome sequences for two affected dogs. The *T* allele of a *PIGN:c.398C* > *T* transition was the only rare, homozygous, predicted amino acid altering sequence variant that was found in both of these animals. All 25 of our *c.398T* allele homozygous DNA samples were from cPxD-affected dogs, whereas none of the 1053 control SCWT and Poodles and none of the 132 genotyped dogs from other breeds were *c.398T* homozygotes. This established a strong association between the clinical disease and *T* allele homozygosity. None of the 15 *PIGN:c.398C/T* heterozygous SCWT were known to have exhibited episodes of dyskinesia, which supports the inheritance of SCWT cPxD as an autosomal recessive trait.

**Fig. 3:**
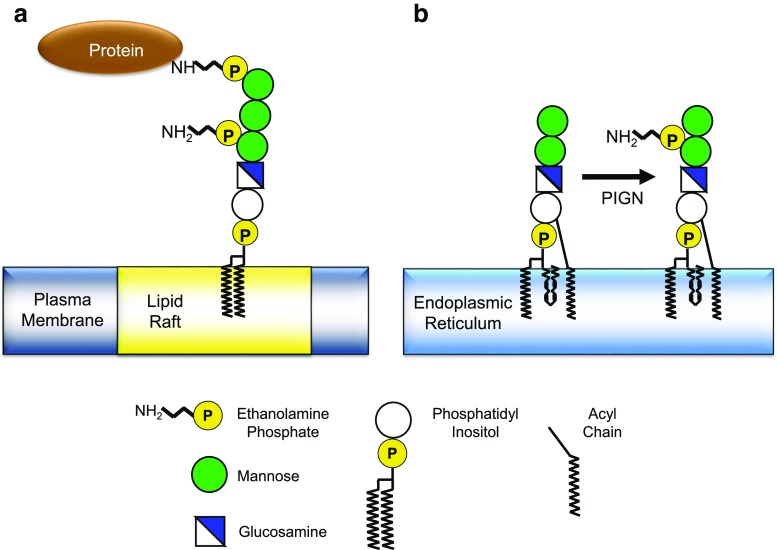
Structure and synthesis of GPI. **a** The core structure of the GPI anchor is a phosphatidylinositol moiety, a glucosamine moiety, three mannoses, and an ethanolamine phosphate (EtNP) on the terminal mannose. The lipid tails of PI are embedded in lipid rafts in the plasma membrane, and cell-surface proteins are bound to the terminal EtNP. Another EtNP bound to the first mannose is found in all mammalian cells. **b** PIGN adds the EtNP to the first mannose during the synthesis of GPI in the endoplasmic reticulum [39]

*PIGN* is 1 of more than 26 genes that encode the polypeptides which contribute to the biosynthesis, protein attachment, and remodeling of GPI anchors. GPI anchors tether a diverse group of cell-surface proteins to the plasma membrane (Fig. 3a). Amide bonds covalently attach the cell-surface protein to an ethanolamine phosphate at the outer end of the GPI anchor, while at the inner end, aliphatic chains intercalate into the plasma membranes and bind to hydrophobic lipid-raft components. A complex core glycolipid provides a covalent bridge between the two ends of the GPI anchor [39, 44]. During GPI assembly in the endoplasmic reticulum, ethanolamine phosphate side chains are added to mannosyl moieties in the core glycolipid. GPI ethanolamine phosphate transferase-1 (PIGN), the enzyme encoded by *PIGN*, facilitates the transfer of an ethanolamine phosphate from phosphatidylethanolamine to the innermost mannosyl moiety in a partially assembled core glycolipid intermediate (Fig. 3b) [45–47]. The existence of *PIGN*-associated deficiency diseases indicates that this ethanolamine phosphate side chain has an important biological function, probably related to its role in the recognition of GPI glycolipids by the transamidase complex that attaches proteins to the GPI anchors [48]. Flow cytometry has been used to demonstrate altered expression of these proteins with mutations of *PIGN* in humans [38, 49–51].

The variant *PIGN:c.398T* allele is predicted to result in a missense mutation, PIGN:p. T133I, in the phosphodiesterase domain. Evidence that the PIGN:p. T133I substitution partially impairs biological function was obtained by transiently transfecting *PIGN-*knockout HEK293 cells with a human *PIGN* cDNA construct designed to encode the p. T133I substitution. As demonstrated by flow cytometry, the human PIGN:p. T133I cDNA was ∼60% as effective as wild-type PIGN cDNA in restoring CD59 surface antigen to the *PIGN-*knockout cells. Available sequence information indicates that *PIGN* orthologs in at least 146 vertebrate species have a threonine codon at the position orthologous to codon 133 in the human and canine *PIGN*; however, at least two vertebrate species, the pig and the cape elephant shrew, have a serine codon at this position (Supplementary Table 2). This suggested that the hydroxyl moiety of threonine or serine might be required for normal biological function. To test this hypothesis, we transiently transfected *PIGN*-knockout HEK293 cells with recombinant human PIGN cDNA designed to encode either PIGN:p. T133S or PIGN:p. T133V. With both of these constructs, the restoration of CD59 surface antigen was similar to that produced by the wild-type PIGN cDNA. The apparently normal function of PIGN with a valine at position 133 indicates that hydroxyl moiety at this position is not a functional requirement. Nonetheless, the contrast in flow cytometry patterns obtained with the various cDNAs substantiates the significance of the 30–40% decrease in CD59 surface antigen restoration that was obtained with the cDNA containing the p. T133I variant.

N-ethyl-N-nitrosourea-derived mice with a homozygous truncating splice-site mutation in *Pign* die in utero with developmental deficiencies including holoprosencephaly [52]. Similarly, a human fetus died with a diaphragmatic hernia and many other severe developmental abnormalities, apparently due to a homozygous nullifying splice-site mutation [53]. Individuals born with other homozygous or compound heterozygous *PIGN* mutations share a developmental deficiency syndrome known as multiple congenital anomalies-hypotonia-seizures syndrome-1 or MCAHS1 (OMIM #614,080). The current literature describes at least 17 MCAHS1 patients from 9 families carrying 13 different *PIGN* mutations [38, 49–51, 54, 55]. These patients had neonatal hypotonia, severe developmental delays, congenital anomalies, visual impairment, hyporeflexia, tremors, and seizures. Some exhibited choreoathetosis and gait abnormalities, while others had structural brain abnormalities including delayed myelination, cortical atrophy, diffusion restriction in the globus pallidi and corticospinal tracts, and cerebellar atrophy or parenchymal loss in the vermis [38, 51, 55]. The severity of clinical signs in MCAHS1 patients appears to correlate with the functional severity of the *PIGN* mutations. The most severe signs, which included multiple structural and functional developmental anomalies, occurred in a neonate with a frameshift mutation in trans with a multi-exon microdeletion. In contrast, a child with likely hypomorphic compound heterozygous missense *PIGN* mutations showed no dysmorphic features, moderate developmental delay, and a later onset of seizures and spastic quadriparesis [55].

The cPxD described in the current report is less severe than the human and murine *PIGN*-associated phenotypes. In contrast to the developmental delays and early mortality seen in humans [38, 51, 55] and mice [52], respectively, with loss of PIGN function, the first episodes of dyskinesia occurred in young-adult dogs. The dogs appeared to be normal prior to the onset of the dyskinesia and between episodes. The marked differences in severity may be due to inherent differences between species. Alternatively, the differences in severity may occur because the canine mutation encodes a product which retains a relatively greater amount of residual functional activity. This second possibility is supported by the finding that transfection of *PIGN-*knockout HEK293 cells with human T133I PIGN cDNA in a strong promoter-driven pME vector fully restored CD59 surface expression, whereas a recent report [38] showed that transfection of *PIGN-*knockout HEK293 cells with the same strong promoter-driven vector and PIGN cDNA carrying the MCAHS1-associated S270P resulted in markedly reduced CD59 surface expression (see Fig. 2b in reference [38]). Thus, it seems possible that hypomorphic mutations of human *PIGN* could also cause PxD or other disease phenotypes that are less severe than those of MCAHS1. Similar variability in phenotype is seen in *SLC2A1* variants associated with PxD in humans [14, 15, 56].

In summary, we have described a paroxysmal dyskinesia in dogs that most closely resembles PNKD but differs from the known human PxDs; in that, it is autosomal recessively inherited. This disease is very likely caused by a hypomorphic missense mutation in *PIGN*, which encodes an enzyme in the biosynthetic pathway for GPI anchors. If so, it expands the phenotypes associated with altered GPI function and suggests candidate genes to investigate in human PxD that have not had a causal mutation identified. The spontaneous canine disease could serve as an animal model to investigate the pathogenesis of PxD and potential therapies. Nonetheless, we cannot rule out the possibility that a variant in tight linkage disequilibrium with the *PIGN:c.398C* > *T* transition is the true cause of the disease. Generation of a mouse model expressing the *PIGN:c.398T* variant that recapitulated the phenotype seen in dogs or the identification of other *PIGN* variants in dogs with cPxD would help confirm that the variant is the causal and permit further studies on its effect on GPI function and the difference in phenotype with the null mutations in mice and humans.

## Electronic supplementary material

Table S1 (DOCX 44 kb)

Table S2 (GIF 3996 kb)

High Resolution Table S2 (TIFF 39334 kb)

Video S1 (WMV 5762 kb)
