# Supplementary material for: A homozygous PIGN missense mutation in Soft-Coated Wheaten Terriers with a canine paroxysmal dyskinesia
Source: Neurogenetics. 2016 Nov 28;18(1):39–47. doi: 10.1007/s10048-016-0502-4 (PMC5243907; doi:10.1007/s10048-016-0502-4)
Supplement: Supplementary file 1 — (DOCX 44 kb) [file 10048_2016_502_MOESM1_ESM.docx]

**Supplemental Table 1a: Homozygous unique coding variants in first whole genome sequence produced with PCR-amplified libraries.**

| GENE | CFA | CHR_POS | REF_BASE | VARIANT | AA_CHANGE | COVERAGE |
| --- | --- | --- | --- | --- | --- | --- |
| *ACCN4* | 37 | 26025744 | A | C | 289D>A | 3 |
| *ACCN4* | 37 | 26025758 | A | C | 294T>P | 4 |
| *ACOXL* | 17 | 35270351 | T | G | 132V>G | 3 |
| *ACOXL* | 17 | 35270356 | C | G | 134L>V | 3 |
| *ADAMTS15* | 5 | 4458444 | T | A | 787K>M | 6 |
| *AHNAK* | 18 | 54109648 | G | A | 3709V>I | 3 |
| *ANXA7* | 4 | 23951430 | C | G | 49A>P | 2 |
| *ANXA7* | 4 | 23951436 | G | A | 47P>S | 2 |
| *ANXA7* | 4 | 23951442 | T | C | 45T>A | 2 |
| *ANXA7* | 4 | 23951448 | C | T | 43G>R | 2 |
| *ARHGAP20* | 5 | 22230860 | C | delCCC | In-Frame | 2 |
| *ASPG* | 8 | 71757312 | T | G | 129V>G | 2 |
| *ATP13A2* | 2 | 81214960 | T | C | 1148F>S | 5 |
| *BAI2* | 2 | 69360924 | A | C | 271N>T | 3 |
| *BAI2* | 2 | 69360926 | T | C | 272S>P | 3 |
| *BEGAIN* | 8 | 68796283 | T | G | 191D>A | 8 |
| *BRWD1* | 31 | 34164606 | C | A | 970G>C | 2 |
| *C10H2orf55* | 10 | 43842573 | A | C | 423T>P | 7 |
| *C14H7orf60* | 14 | 52424229 | G | A | 151T>I | 2 |
| *C17H1orf56* | 17 | 60135634 | A | C | 58K>Q | 8 |
| *C20H19orf29* | 20 | 55815800 | T | C | 582S>P | 5 |
| *C30H15orf61* | 30 | 31644657 | A | C | 114N>T | 5 |
| *C37H2orf47* | 37 | 9138416 | T | G | 226C>G | 2 |
| *CADM2* | 31 | 1965176 | C | T | 297E>K | 8 |
| *CAPZA2* | 14 | 55809759 | C | G | 129R>G | 4 |
| *CCDC40* | 9 | 1641190 | G | C | 338A>G | 3 |
| *CCNDBP1* | 30 | 10054083 | A | C | 39E>A | 6 |
| *CD14* | 2 | 35792097 | C | A | 156G>W | 40 |
| *CDK18* | 38 | 1999372 | A | C | 448Q>H | 2 |
| *CEP135* | 13 | 48248017 | A | G | 437D>G | 5 |
| *CNO* | 3 | 58822876 | T | G | 156V>G | 6 |
| *CNO* | 3 | 58822878 | T | G | 157Y>D | 6 |
| *CRBN* | 20 | 14247670 | G | A | 142E>K | 3 |
| *CSNK2A2* | 2 | 58398399 | C | G | 5P>A | 2 |
| *CSNK2A2* | 2 | 58398400 | C | G | 5P>R | 2 |
| *CSNK2A2* | 2 | 58398402 | C | G | 6P>A | 2 |
| *CSNK2A2* | 2 | 58398403 | C | G | 6P>R | 2 |
| *CYP11B2* | 13 | 36877031 | A | C | 239Y>S | 3 |
| *DCTN6* | 16 | 34124786 | G | T | 97Q>K | 4 |
| GENE | **CFA** | **CHR_POS** | **REF_BASE** | **VARIANT** | **AA_CHANGE** | **COVERAGE** |
| *DCTN6* | 16 | 34124789 | A | G | 96S>P | 4 |
| *DIRC2* | 33 | 25910104 | T | G | 298S>A | 2 |
| *DLA-12* | 12 | 933665 | G | A | 58D>N | 3 |
| *DLL1* | 12 | 72261728 | T | C | 1204X>W | 3 |
| *DLL1* | 12 | 72261743 | A | G | 1199M>T | 3 |
| *EBF1* | 4 | 51381583 | A | G | 78Q>R | 8 |
| *EBF1* | 4 | 51381586 | G | C | 79G>A | 8 |
| *EID1* | 30 | 15109191 | C | A | 10L>M | 2 |
| *EID1* | 30 | 15109274 | C | G | 37S>R | 7 |
| *ESD* | 22 | 4569705 | T | C | 264F>L | 3 |
| *FAF1* | 15 | 10425121 | G | C | 144W>S | 5 |
| *FAM120C* | 39 | 45943538 | T | G | 7K>Q | 2 |
| *FAM120C* | 39 | 45943539 | T | G | 6K>N | 2 |
| *FAM120C* | 39 | 45943540 | T | A | 6K>I | 2 |
| *FAM120C* | 39 | 45943541 | T | A | 6K>X | 2 |
| *FAM120C* | 39 | 45943542 | T | G | 5E>D | 2 |
| *FAM120C* | 39 | 45943543 | T | C | 5E>G | 2 |
| *FAM131A* | 34 | 17222704 | C | insG | FS | 2 |
| *FAM131A* | 34 | 17222709 | T | G | 405L>R | 2 |
| *FAM98A* | 17 | 26677147 | G | C | 236S>C | 4 |
| *FBRSL1* | 26 | 536801 | T | G | 770D>A | 9 |
| *FFAR1* | 1 | 117257822 | T | G | 39T>P | 3 |
| *FRYL* | 13 | 44398730 | T | C | 664D>G | 6 |
| *FRYL* | 13 | 44398733 | T | C | 663E>G | 6 |
| *FTH1* | 11 | 39037568 | A | G | 14H>R | 3 |
| *GALNT13* | 36 | 973660 | T | C | 294L>P | 2 |
| *GEM* | 29 | 39025840 | A | G | 241L>P | 2 |
| *GLB1* | 23 | 3769351 | A | G | 276D>G | 6 |
| *GPAA1* | 13 | 37579195 | C | G | 394I>M | 2 |
| *GPAA1* | 13 | 37579198 | T | G | 395H>Q | 2 |
| *GPAA1* | 13 | 37579201 | C | G | 396F>L | 2 |
| *GPRASP1* | 39 | 76315045 | C | G | 436R>G | 7 |
| *GPRASP1* | 39 | 76315046 | G | A | 436R>Q | 7 |
| *GPRASP1* | 39 | 76315047 | G | insA | FS | 7 |
| *GPRASP1* | 39 | 76315048 | T | A | 437W>R | 7 |
| *GPRASP1* | 39 | 76315049 | G | A | 437W>X | 7 |
| *GPRASP1* | 39 | 76315055 | A | T | 439D>V | 7 |
| *HDAC10* | 10 | 17005973 | T | C | 735S>P | 8 |
| *HDAC10* | 10 | 17005979 | A | C | 737T>P | 8 |
| *HMP19* | 4 | 38493737 | T | G | 136I>L | 10 |
| *HOXC13* | 27 | 1322988 | A | G | 128L>P | 3 |
|  |  |  |  |  |  |  |
| GENE | **CFA** | **CHR_POS** | **REF_BASE** | **VARIANT** | **AA_CHANGE** | **COVERAGE** |
| *HR* | 25 | 35131009 | C | insG | FS | 6 |
| *HR* | 25 | 35131010 | A | G | 847Q>R | 6 |
| *HR* | 25 | 35131013 | A | G | 848E>G | 6 |
| *IL17B* | 4 | 59583768 | G | C | 115D>H | 8 |
| *IRF2BP1* | 1 | 109740090 | C | insG | FS | 2 |
| *ITGB3BP* | 5 | 46682272 | A | G | 19N>D | 2 |
| *KCNA4* | 21 | 50116932 | A | C | 192V>G | 4 |
| *KCNA4* | 21 | 50116935 | A | C | 191V>G | 4 |
| *KCNF1* | 17 | 7752774 | C | G | 42L>V | 2 |
| *KIAA1109* | 19 | 17956232 | T | A | 1069E>V | 5 |
| *KIAA1109* | 19 | 17956233 | C | A | 1069E>X | 5 |
| *KIF20B* | 28 | 4621592 | A | G | 764T>A | 5 |
| *KIF26A* | 8 | 71822968 | T | C | 72L>P | 3 |
| *LAMA4* | 12 | 68574102 | T | G | 672H>P | 5 |
| *LOC100682952* | 10 | 8635905 | T | C | 1M>V | 3 |
| *LOC100683298* | 20 | 54432891 | T | C | 195S>P | 5 |
| *LOC100683298* | 20 | 54432895 | A | C | 196N>T | 5 |
| *LOC100685794* | 5 | 36098038 | T | A | 3M>K | 2 |
| *LOC100688783* | 26 | 27216883 | A | T | 92T>S | 9 |
| *LOC100688783* | 26 | 27216904 | G | A | 99E>K | 9 |
| *LOC100855692* | 10 | 61863067 | A | G | 333I>T | 2 |
| *LOC100855864* | 8 | 72736858 | T | G | 108S>A | 9 |
| *LOC100856304* | 2 | 18383718 | T | G | 74D>A | 2 |
| *LOC100856304* | 2 | 18383724 | A | T | 72L>H | 2 |
| *LOC100856304* | 2 | 18383729 | A | C | 70F>L | 2 |
| *LOC100856351* | 11 | 39043361 | A | G | 81C>R | 2 |
| *LOC477695* | 27 | 35133108 | C | T | 142H>Y | 2 |
| *LOC487073* | 2 | 1320994 | T | G | 864V>G | 6 |
| *LOC487543* | 30 | 13354528 | C | G | 121A>G | 2 |
| *LOC487670* | 30 | 39945445 | C | G | 535A>P | 6 |
| *LOC487815* | 32 | 1475718 | T | G | 273Q>P | 5 |
| *LOC490390* | 7 | 39229174 | A | G | 235E>G | 8 |
| *LOC490390* | 7 | 39229176 | C | G | 236Q>E | 8 |
| *LOC607594* | 25 | 24797574 | A | G | 51E>G | 5 |
| *LOC607594* | 25 | 24797576 | C | G | 52R>G | 5 |
| *LOC610891* | 38 | 23651499 | T | G | 896V>G | 2 |
| *LOC611902* | 17 | 37499510 | A | G | 116N>D | 2 |
| *LOC611902* | 17 | 37499511 | A | G | 116N>S | 2 |
| *LOC611902* | 17 | 37499513 | T | A | 117L>I | 2 |
| *LONRF3* | 39 | 91106869 | A | G | 125E>G | 6 |
| *LPHN1* | 20 | 48380578 | C | G | 1445P>A | 2 |
| *LPHN1* | 20 | 48380578 | C | G | 1445P>A | 2 |
| GENE | **CFA** | **CHR_POS** | **REF_BASE** | **VARIANT** | **AA_CHANGE** | **COVERAGE** |
| *LRPPRC* | 10 | 46438325 | T | G | 6R>S | 2 |
| *LRRC27* | 28 | 40298617 | A | G | 858E>G | 6 |
| *LRRC33* | 33 | 29699324 | C | G | 682P>A | 6 |
| *LRRC45* | 9 | 298900 | T | G | 365D>A | 2 |
| *MAPKAPK2* | 7 | 5901211 | A | C | 241T>P | 9 |
| *MBIP* | 8 | 14886709 | T | C | 322S>G | 2 |
| *MEAF6* | 15 | 4947987 | T | G | 81V>G | 6 |
| *MRGPRG* | 18 | 47136179 | C | G | 100R>P | 10 |
| *MRPL46* | 3 | 51702661 | T | G | 184T>P | 3 |
| *MYO5A* | 30 | 18052103 | C | G | 952R>P | 2 |
| *MYO5A* | 30 | 18052106 | G | C | 951A>G | 2 |
| *NF1* | 9 | 41505776 | T | C | 2212D>G | 5 |
| *NFKBIZ* | 33 | 8125937 | A | G | 72K>R | 2 |
| *NFKBIZ* | 33 | 8125951 | T | C | 77S>P | 3 |
| *NFKBIZ* | 33 | 8125958 | T | C | 79F>S | 3 |
| *NFKBIZ* | 33 | 8125965 | G | C | 81R>S | 3 |
| *NFKBIZ* | 33 | 8125967 | G | C | 82G>A | 3 |
| *NID1* | 4 | 3985012 | C | G | 720S>R | 3 |
| *NID1* | 4 | 3985014 | A | C | 721H>P | 3 |
| *NUDT6* | 19 | 17375074 | A | G | 131E>G | 3 |
| *PALM* | 20 | 57855581 | T | G | 144T>P | 7 |
| *PARP14* | 33 | 25782161 | A | C | 908E>A | 8 |
| *PIGN* | 1 | 14705240 | C | T | 133T>I | 16 |
| *PKHD1L1* | 13 | 10054619 | T | G | 3805C>W | 11 |
| *PKHD1L1* | 13 | 10054621 | C | G | 3806A>G | 11 |
| *PLBD2* | 26 | 10709971 | C | A | 134L>I | 2 |
| *PLBD2* | 26 | 10709975 | T | A | 135F>Y | 2 |
| *PLBD2* | 26 | 10709977 | T | A | 136L>M | 2 |
| *PLBD2* | 26 | 10709981 | C | A | 137P>H | 2 |
| *PLEC* | 13 | 37450827 | T | G | 4573Y>S | 5 |
| *PML* | 30 | 37265646 | C | T | 500R>W | 47 |
| *PPM1E* | 9 | 33350900 | G | A | 96G>D | 3 |
| *PRR12* | 1 | 106921117 | A | C | 1554C>G | 7 |
| *PRSS33* | 6 | 38320579 | G | C | 43R>G | 6 |
| *RAP1A* | 17 | 63742031 | C | A | 25Q>H | 2 |
| *RAP1A* | 17 | 63742032 | T | G | 25Q>P | 2 |
| *RBM10* | 39 | 40762898 | G | C | 145G>R | 2 |
| *RBM10* | 39 | 40762904 | G | C | 147G>R | 2 |
| *RBM10* | 39 | 40762907 | G | C | 148G>R | 2 |
| *RBM10* | 39 | 40762913 | G | C | 150G>R | 2 |
| *RFWD2* | 7 | 23321045 | A | G | 114K>E | 5 |
| *RIN2* | 24 | 3371828 | T | G | 387K>N | 9 |
| GENE | **CFA** | **CHR_POS** | **REF_BASE** | **VARIANT** | **AA_CHANGE** | **COVERAGE** |
| *RNASEH2B* | 22 | 967992 | G | insA | FS | 6 |
| *ROR2* | 1 | 95278502 | C | G | 464A>G | 3 |
| *RRP1B* | 31 | 37663897 | G | insT | FS | 2 |
| *RSPH4A* | 1 | 57074175 | T | A | 572D>E | 3 |
| *RXFP3* | 4 | 73873384 | A | C | 243V>G | 4 |
| *S100PBP* | 2 | 68499452 | T | G | 400T>P | 3 |
| *SCNN1B* | 6 | 22462890 | C | G | 344A>P | 5 |
| *SCNN1B* | 6 | 22462892 | T | G | 343N>T | 5 |
| *SEPT1* | 6 | 17708699 | A | G | 117R>G | 4 |
| *SEPT1* | 6 | 17708702 | C | G | 118P>A | 4 |
| *SLC12A3* | 2 | 59437916 | T | C | 496K>E | 12 |
| *SLC25A46* | 3 | 1718383 | A | C | 35L>R | 4 |
| *SLC25A46* | 3 | 1718389 | C | A | 33G>V | 4 |
| *SLC25A46* | 3 | 1718392 | C | A | 32G>V | 4 |
| *SLC25A46* | 3 | 1718393 | C | T | 32G>R | 4 |
| *SLC29A3* | 4 | 22098019 | A | insC | FS | 2 |
| *SLC30A1* | 7 | 9959075 | C | G | 104Q>H | 2 |
| *SLC35F5* | 19 | 35270909 | C | A | 98N>K | 5 |
| *SLK* | 28 | 16312240 | T | C | 146L>S | 2 |
| *SMAD6* | 30 | 31013728 | A | C | 485T>P | 5 |
| *SMAD6* | 30 | 31013731 | T | C | 486S>P | 5 |
| *SOX30* | 4 | 52587962 | A | G | 203D>G | 10 |
| *SP7* | 27 | 1886597 | G | C | 341E>Q | 3 |
| *SPACA1* | 12 | 47450050 | C | G | 19L>V | 13 |
| *SPATA21* | 2 | 81306486 | G | C | 433E>Q | 4 |
| *SPEN* | 2 | 81687640 | A | C | 3227V>G | 4 |
| *SSC5D* | 1 | 102196945 | G | insA | FS | 2 |
| *SSC5D* | 1 | 102196950 | C | delC | FS | 2 |
| *STOML1* | 30 | 37226705 | C | T | 127V>M | 23 |
| *SYNJ1* | 31 | 27356222 | G | A | 412A>V | 6 |
| *TAF6L* | 18 | 53899260 | T | G | 469T>P | 5 |
| *TAOK1* | 9 | 43594263 | A | C | 175M>L | 6 |
| *TBXA2R* | 20 | 55824993 | C | G | 195A>G | 2 |
| *TENC1* | 27 | 2099162 | C | G | 1053E>D | 2 |
| *TGDS* | 22 | 45386237 | T | G | 295R>S | 3 |
| *TGFA* | 10 | 68910846 | C | G | 422V>L | 5 |
| *THEM4* | 17 | 60925021 | T | G | 24H>P | 3 |
| *TJP3* | 20 | 55722132 | T | G | 789D>A | 4 |
| *TMCC2* | 38 | 1771057 | G | C | 443A>P | 13 |
| *TMEM151A* | 18 | 51010388 | G | C | 145A>G | 4 |
| *TMPRSS9* | 20 | 56680769 | A | G | 310L>P | 2 |
| *TOMM20* | 4 | 4800927 | C | T | 136A>V | 2 |
| GENE | **CFA** | **CHR_POS** | **REF_BASE** | **VARIANT** | **AA_CHANGE** | **COVERAGE** |
| *TRIM23* | 2 | 51353677 | T | G | 61T>P | 3 |
| *TRIOBP* | 10 | 26884008 | C | A | 1584A>S | 12 |
| *TRIOBP* | 10 | 26884010 | C | G | 1583G>A | 12 |
| *TRIOBP* | 10 | 26884011 | C | T | 1583G>S | 12 |
| *TRIOBP* | 10 | 26884028 | G | A | 1577P>L | 2 |
| *TRMT1* | 20 | 49101235 | C | G | 29Q>E | 11 |
| *UCHL1* | 3 | 71410964 | A | T | 3L>H | 2 |
| *ULK3* | 30 | 37904557 | A | G | 23L>P | 6 |
| *USP25* | 31 | 12652955 | A | G | 722R>G | 3 |
| *USP34* | 10 | 61289706 | C | G | 2885A>P | 5 |
| *UTRN* | 1 | 35876816 | A | C | 750Q>H | 3 |
| *VANGL2* | 38 | 21828498 | C | G | 89G>R | 10 |
| *VANGL2* | 38 | 21828499 | C | G | 88K>N | 10 |
| *VANGL2* | 38 | 21828500 | T | C | 88K>R | 10 |
| *VIM* | 2 | 19671672 | C | T | 457E>K | 5 |
| *WDR36* | 3 | 1490475 | T | C | 75R>G | 5 |
| *WNT5B* | 27 | 43670032 | C | A | 168Y>X | 3 |
| *YARS2* | 27 | 16139787 | G | A | 97G>S | 9 |
| *YWHAE* | 9 | 45512480 | T | C | 235M>V | 3 |
| *ZBTB4* | 5 | 32388310 | T | C | 577I>V | 2 |
| *ZC3H6* | 17 | 36646542 | C | A | 67H>N | 2 |
| *ZC3H6* | 17 | 36646543 | A | G | 67H>R | 2 |
| *ZC3H6* | 17 | 36646544 | C | A | 67H>Q | 2 |

**Supplemental Table 1b: Homozygous unique coding variants in second whole genome sequence produced with PCR-free libraries.**

| GENE | CFA | CHR_POS | REF_BASE | | VARIANT | AA_CHANGE | COVERAGE |
| --- | --- | --- | --- | --- | --- | --- | --- |
| *ACTR5* | 24 | 27154461 | C | G | | 55A>G | 3 |
| *ACTR5* | 24 | 27154463 | T | G | | 56C>G | 3 |
| *ADAMTS19* | 11 | 18273233 | G | delG | | FS | 2 |
| *APOB* | 17 | 15888601 | G | A | | 1560T>M | 19 |
| *ARHGEF26* | 23 | 48250284 | G | C | | 20R>P | 3 |
| *ARHGEF26* | 23 | 48250289 | G | A | | 22G>S | 3 |
| *ATP8B3* | 20 | 57160284 | G | C | | 1394R>S | 10 |
| *C18H11orf35* | 18 | 25664137 | C | G | | 739G>R | 3 |
| *C28H10orf82* | 28 | 27224783 | G | T | | 152Y>X | 28 |
| *C30H15orf39* | 30 | 38175075 | C | delCCGGCA | | In-Frame | 11 |
| *C4H1orf96* | 4 | 9878498 | G | delGAGGCGGCG | | In-Frame | 8 |
| *CABLES2* | 24 | 46375466 | T | G | | 138T>P | 4 |
| *CNNM3* | 10 | 45154194 | G | C | | 147P>R | 2 |
| *CSN1S1* | 13 | 59430621 | T | G | | 153D>E | 26 |
| *DFNB31* | 11 | 68699676 | C | delC | | FS | 2 |
| *ECHDC2* | 5 | 55972146 | G | A | | 48R>Q | 2 |
| *FAM45A* | 28 | 29346298 | A | G | | 6S>G | 2 |
| *FAM45A* | 28 | 29346301 | G | insG | | FS | 2 |
| *FAM73A* | 6 | 69026458 | G | A | | 527L>F | 20 |
| *FBXO45* | 33 | 29635933 | A | delAT | | FS | 2 |
| *FBXO45* | 33 | 29635937 | T | insT | | FS | 2 |
| *GPR83* | 21 | 6536825 | C | delCTT | | In-Frame | 2 |
| *GTF3C1* | 6 | 19168295 | G | A | | 1639E>K | 13 |
| *JMJD8* | 6 | 39891999 | G | delGTGGCGCCGGTGCTGC | | FS | 4 |
| *KCNQ3* | 13 | 29062325 | T | G | | 16N>T | 3 |
| *KIF12* | 11 | 68368122 | G | A | | 81A>V | 13 |
| *LATS1* | 1 | 40446218 | T | C | | 345I>V | 11 |
| *LEPREL2* | 27 | 38194601 | G | C | | 101S>W | 3 |
| *LOC100682747* | 14 | 11647458 | G | C | | 182E>Q | 8 |
| *LOC100682857* | 16 | 22212954 | A | T | | 235H>L | 2 |
| *LOC100682857* | 16 | 22212957 | T | delTCG | | In-Frame | 2 |
| *LOC100684233* | 12 | 36497100 | T | delTTTTT | | Splice | 7 |
| *LOC100684399* | 35 | 765733 | G | A | | 254G>R | 5 |
| *LOC100688053* | 6 | 53880544 | C | insT | | FS | 14 |
| *LOC100855863* | 38 | 22151133 | C | G | | 589A>G | 4 |
| *LOC475279* | 14 | 45015191 | A | C | | 445V>G | 4 |
| *LOC484210* | 1 | 98449334 | G | delG | | FS | 4 |
| *LOC484210* | 1 | 98455777 | C | G | | 1228A>P | 3 |
| LOC485980 | 24 | 47296889 | C | delCC | | FS | 3 |
| GENE | **CFA** | **CHR_POS** | **REF_BASE** | **VARIANT** | | **AA_CHANGE** | **COVERAGE** |
| *LOC489893* | 6 | 14726554 | C | G | | 1164R>P | 2 |
| *LOC610174* | 15 | 12776779 | A | delA | | FS | 2 |
| *LOC610174* | 15 | 12776785 | A | delAGACGG | | In-Frame | 2 |
| *LOC612378* | 6 | 57025306 | C | G | | 147S>R | 2 |
| *LOC612378* | 6 | 57025307 | T | A | | 148S>T | 2 |
| *LOR* | 17 | 61995003 | T | G | | 229S>A | 4 |
| *LOR* | 17 | 61995004 | C | G | | 229S>C | 4 |
| *LOR* | 17 | 61995153 | T | G | | 279C>G | 2 |
| *MAP3K15* | 39 | 15465777 | T | C | | 29E>G | 2 |
| *MAST3* | 20 | 44878332 | A | C | | 1M>R | 4 |
| *MIER2* | 20 | 58064321 | C | A | | 245R>S | 6 |
| *ODZ1* | 39 | 96111976 | A | G | | 193L>P | 3 |
| *PHLPP1* | 1 | 14234138 | T | G | | 31E>A | 2 |
| *PIGN* | 1 | 14705240 | C | T | | 133T>I | 16 |
| *PKD1L3* | 5 | 77754396 | G | delG | | FS | 23 |
| *PLK5* | 20 | 57343915 | A | G | | 11S>P | 5 |
| *PRDM12* | 9 | 53284486 | G | insGCG | | In-Frame | 5 |
| *PRR24* | 1 | 108705031 | T | delT | | FS | 3 |
| *PRR24* | 1 | 108705036 | T | C | | 20N>S | 3 |
| *RAB12* | 7 | 74780877 | G | A | | 87A>T | 12 |
| *RIN3* | 8 | 1777689 | C | T | | 319P>L | 5 |
| *S1PR5* | 20 | 50644336 | G | C | | 240A>P | 2 |
| *SHISA2* | 25 | 13472951 | A | delA | | FS | 2 |
| *SHISA2* | 25 | 13472952 | A | T | | 382N>I | 2 |
| *TMEM70* | 29 | 22480075 | T | G | | 105C>G | 6 |
| *TRIM44* | 18 | 32351165 | G | delGGGCGG | | In-Frame | 6 |
| *TTC39A* | 15 | 9939623 | A | C | | 285Y>S | 4 |
| *ZMIZ2* | 99 | 264737 | G | insC | | FS | 2 |
