# Supplementary figures and images for: A homozygous PIGN missense mutation in Soft-Coated Wheaten Terriers with a canine paroxysmal dyskinesia
Source: Neurogenetics. 2016 Nov 28;18(1):39–47. doi: 10.1007/s10048-016-0502-4 (PMC5243907; doi:10.1007/s10048-016-0502-4)

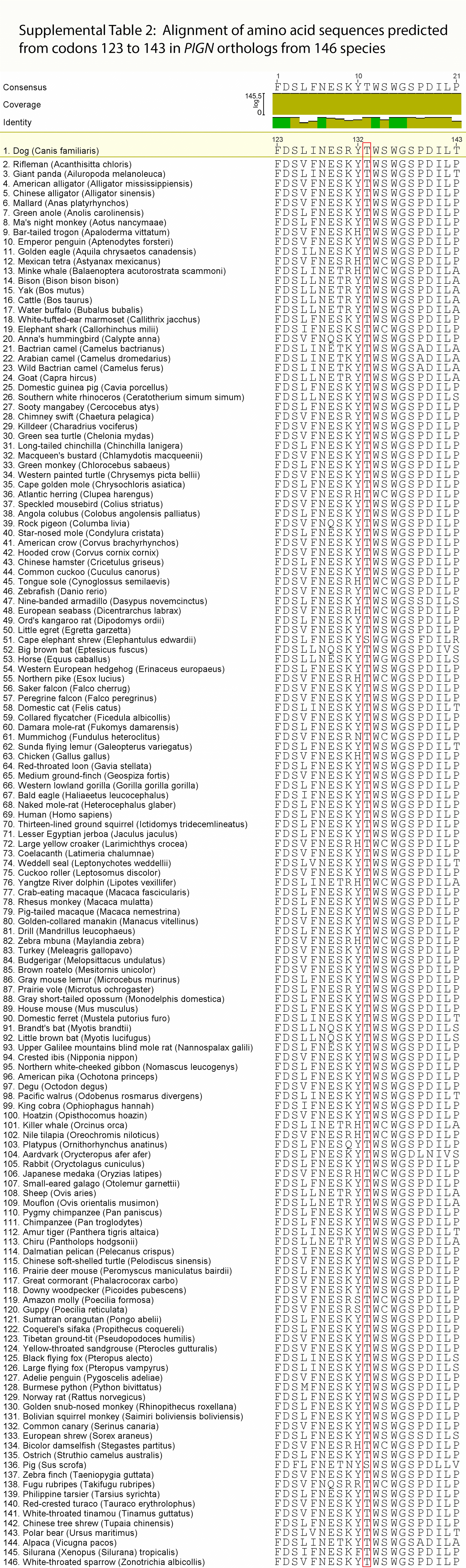

Supplement: Supplementary file 2 — (GIF 3996 kb) [file 10048_2016_502_Fig4_ESM.gif]

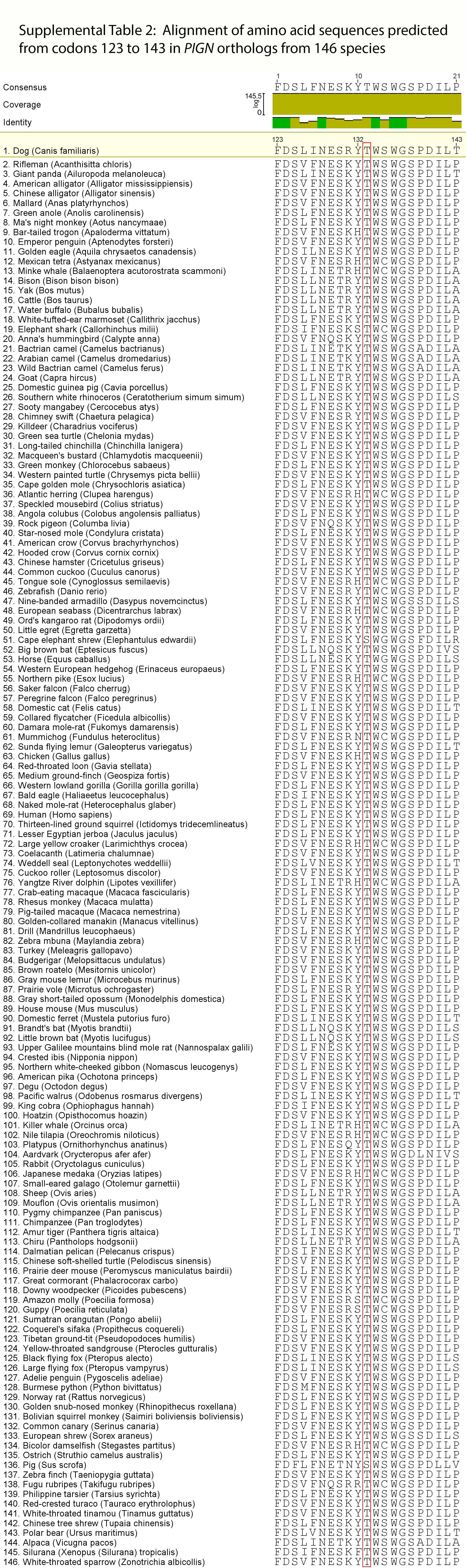

Supplement: Supplementary file 3 — (TIFF 39334 kb) [file 10048_2016_502_MOESM2_ESM.tif]
